# The perception of affective touch in women affected by obesity

**DOI:** 10.3389/fpsyg.2023.1171070

**Published:** 2023-08-28

**Authors:** Sofia Tagini, Massimo Scacchi, Alessandro Mauro, Federica Scarpina

**Affiliations:** ^1^“Rita Levi Montalcini” Department of Neurosciences, University of Turin, Turin, Italy; ^2^Istituto Auxologico Italiano, I.R.C.C.S., U.O. di Neurologia e Neuroriabilitazione, Ospedale San Giuseppe, Piancavallo, Italy; ^3^Istituto Auxologico Italiano, I.R.C.C.S., U.O. Medicina Generale, Ospedale San Giuseppe, Piancavallo, Italy; ^4^Department of Clinical Sciences and Community Health, University of Milan, Milan, Italy

**Keywords:** affective touch, obesity, interpersonal relationships, interpersonal pleasure, imagery

## Abstract

**Introduction:**

Pleasant and comforting bodily contacts characterized intimate and affective interactions. Affective touch informs us about others’ emotions and intentions, sustains intimacy and closeness, protecting from loneliness and psychological distress. Previous evidence points to an altered experience of affective touch in clinical populations reporting interpersonal difficulties. However, there is no investigation of affective touch in obesity, which is often associated with negative affective-relational experiences since childhood.

**Methods:**

This study aimed to provide the first evidence about the experience of affective touch in obesity by comparing 14 women with obesity with 14 women with healthy weight. Participants rated the pleasantness of both imagined and actual tactile stimuli, which consisted of (*i*) soft-brush strokes, (*ii*) touches of the experimenter’s hand, and (*iii*) of a plastic stick (as control, non-affective, stimulation). Participants should report the pleasantness of each kind of touch. Moreover, we explored lifespan experiences of affective touch and interpersonal pleasure in social contexts through self-report questionnaires.

**Results:**

No differences emerged for the pleasantness of affective touch (in both the real and imagery task) between the two groups. However, participants with obesity reported less frequent and less satisfying early experiences of affective touch when compared with the controls.

**Discussion:**

Our results spoke in favor of a preserved experience of affective touch when experimentally probed in obesity, despite a limited early exposure to bodily affective contacts. We interpreted our results in the light of the *social reconnection hypothesis*. Nevertheless, we provided crucial methodological considerations for future research, considering that both the experimenter’s and the brush touch may not resemble adequately real-life experiences, in which affective touch involves intimate people.

## 1. Introduction

Physical contact is a key component of intimate relationships, which typically encompass tender bodily interactions: we refer to this pleasant, gentle, and slow touch as *affective* touch. Both newborns and caregivers reciprocally experience positive emotions during physical interactions, such as comfort and reassurance, which motivate proximity and satisfy both survival and primary relational needs (Bowlby, 1979). Tender mother-infant bodily contacts even trigger the tuning of neural brain activity in the dyad (Nguyen et al., 2021), promoting social bonding and children’s learning (e.g., word learning; Wass et al., 2020). In adulthood, the pleasure experienced in bodily affective interactions motivates individuals to establish and nurture affective relationships (Gallace and Spence, 2016), fulfilling the humans’ fundamental need to belong (Baumeister and Leary, 1995). On the other hand, interpersonal difficulties seem associated with a meager experience of affective touch, pointing out how crucial bodily affective interactions are in promoting satisfying and functional affective relationships. For example, individuals with high social anxiety are not prone to interpersonal interactions involving touch (Wilhelm et al., 2001), whereas an altered hedonic experience of affective touch was reported in clinical populations characterized by atypical relational experiences, such as autism spectrum disorders (Voos et al., 2013; Kaiser et al., 2016; Perini et al., 2021) and anorexia nervosa (Crucianelli et al., 2016, 2021; Davidovic et al., 2018), although Tagini et al. (2023) reported no such alteration. Deprivation of intimate touch was related to anxiety, depression, loneliness, and psychopathology (Floyd, 2014; von Mohr et al., 2021); in turn, the lower the exposure to affective touch across the lifespan (Sailer and Ackerley, 2019; Beltrán et al., 2020) the less its appreciation. On one side, the experience of satisfying and pleasant affective bodily interactions supports social engagement and promotes individuals’ psychological wellbeing; nonetheless, the quality of our relational experience affects individuals’ attitude to intimate bodily interactions.

Therefore, the recent and growing interest in the study of humans’ experience of affective touch, and specifically in the context of psychopathology, may not be surprising. However, we could not find any study exploring this topic in obesity. This omission may be related to the deep-rooted tendency to look at obesity focusing on its physical and health-related consequences, as well as in terms of non-appropriate eating and lifestyle habits. More recently, research in the field of obesity has focused on body from a neuropsychological perspective (Serino et al., 2016; Scarpina et al., 2017, 2022; Tagini et al., 2020a,b), highlighting the importance of investigating how obesity may impact on the cognitive representation of the body and the processing of sensory-motor information. Nevertheless, the effect of obesity goes beyond physical appearance and body perception since it impacts individuals’ wellbeing and social interactions. Individuals who suffer from obesity are less likely to have robust social relationships: lack of self-confidence and the experience of a weight-related stigma make it harder to establish and maintain significant relationships, increasing the risk of gaining weight and high psychological distress (for a review Albano et al., 2019). A developmental onset and subsequent refractory maintenance of obesity were associated with inadequate family communication and low cohesion, family conflicts (Halliday et al., 2014), perceived maternal rejection (Senese et al., 2020), and overall poor family functioning (Warnick et al., 2019). Likely, unresponsive caregivers and weak affective bounds limited the early experience of tender and reassuring bodily interactions, hampering a fulfilling experience of intimate bodily interactions in adulthood.

From a clinical perspective, the understanding of how interpersonal difficulties might interplay with the processing of bodily sensory information (e.g., as reported in anorexia nervosa by Tagini et al., 2023 and autism spectrum disorders by Voos et al., 2013; Kaiser et al., 2016; Perini et al., 2021) may contribute to rehabilitative treatments aimed at restoring the putative role of (affective) touch in communicating and managing emotional distress, reducing emotionally-related overeating behaviors and promoting social cognition. Primarily, this study aimed to provide the first experimental evidence on the processing of affective touch in women with obesity compared to healthy weight women, using the experimental paradigm formerly presented in Tagini et al. (2023). In this procedure, participants rated the pleasantness associated with gentle tactile sensations, consisting of both soft brush strokes (as done traditionally, Essick et al., 1999; Löken et al., 2009; Gordon et al., 2013) and the caressing touch of another human being, as a novel and more veridical interactive scenario (i.e., if compared to a tool-to-body interaction); the touch of a plastic stick was then included as control, neutral, condition of stimulation. Crucially, participants not only judged the pleasantness of touches delivered on their skin but also of corresponding *imagined* tactile stimulations. Since both supportive (e.g., Bussolaro et al., 2012; Falling and Mani, 2016) and not confirmative (Scarpina et al., 2021; Tagini et al., 2021) evidence was reported in the literature about a possible alteration of primary (bottom-up) tactile processing in obesity the evaluation of *imagined* tactile sensations should isolate the potential contribution of an altered *hedonic* (top-down), rather than sensory, processing of affective touch.

Additionally, we aimed to explore whether the processing of bodily sensory information (i.e., affective touch) in the experimental setting was related to participants’ real-life experience of interpersonal bodily contact and social interactions. To this purpose, we included a self-reported evaluation of the experience of affective touch across the lifespan (i.e., *Tactile Biography* questionnaire, Beltrán et al., 2020) and the hedonic value attributed to daily social interactions (i.e., *Anticipatory and Consummatory Interpersonal Pleasure Scale*, ACIPS, Gooding and Pflum, 2014), exploring whether these measures were associated with the pleasure experienced during affective-like bodily interactions in the experimental setting.

In line with the available evidence in other clinical conditions (Voos et al., 2013; Crucianelli et al., 2016, 2021; Kaiser et al., 2016; Davidovic et al., 2018; Perini et al., 2021), we might expect an atypical processing of affective tactile stimuli in obesity, especially in those participants who experienced inadequate and unsupportive caring parental relationships early in life and aversion to social interactions in adulthood. Furthermore, we suggest that the evidence of an altered experience of affective touch in obesity in the case of (also) imagined touches will support the specific involvement of the hedonic (i.e., top-down) than purely sensory (i.e., bottom-up) component of affective touch processing.

## 2. Methods

The study was approved by the ethical committee of the I.R.C.C.S. Istituto Auxologico Italiano and performed in compliance with international ethical standards (World Medical Association, 2013). Naïve volunteers gave their informed written consent before participating in the study; they were free to withdraw at any time of the experimental procedure. All measures, manipulations, and exclusions are acknowledged.

### 2.1. Participants

Right-handed women with obesity participated in the study during the first week of a diagnostic and rehabilitative recovery at the I.R.C.C.S. Istituto Auxologico Italiano, Ospedale San Giuseppe (Piancavallo, VB, Italy). Inclusion criteria were (*i*) a body mass index (BMI) higher than 30 kg/m^2^, which is the critical cut-off for obesity agreed internationally (World Health Organization, 2020) and (*ii*) female gender, considering that gender affects both the perception of affective touch (Russo et al., 2020), the obesity-related phenotype (Legato, 1997; Borders et al., 2006; Kanter and Caballero, 2012) and psychological functioning (Hyde, 2014). Crucially, also the experimenter was always a woman.

Right-handed women with a healthy weight (i.e., with a BMI lower than 30 kg/m^2^; World Health Organization, 2020) and no self-reported history of eating disorders were recruited as controls outside the hospital, through personal contacts of the researchers and word-of-mouth.

In both groups, neurological signs, or symptoms (especially of neuropathic pain), psychiatric comorbidities, or personality disorders (First and Gibbon, 2004) were reasons for exclusion.

### 2.2. Materials and measures

#### 2.2.1. Affective touch paradigm

The experimental tasks adopted in this study overlap the procedure we recently developed to investigate the experience of affective touch in a different clinical population (i.e., anorexia nervosa; Tagini et al., 2023). Tactile stimuli consist of (*i*) gentle strokes of a soft cosmetic brush (Löken et al., 2009; Gordon et al., 2013) and (*ii*) the caressing touch of the experimenter’s hand; the touch of (*iii*) a plastic stick with a rounded tip was taken as a control condition of stimulation (Tagini et al., 2023). We adopted two speeds of stimulation, which differently elicit the C-tactile (CT) fibers deputed to the processing of the *hedonic* dimension of touch (Olausson et al., 2016).

Slow tactile stimuli delivered at 3 cm/s should optimize the response of the CT system, inducing the pleasant sensation typically associated with *affective* touch. Each trial of stimulation last 3 s and consists of one proximal-to-distal 9 cm-stimulation. On the other hand, *non-affective* stimuli were delivered at non-optimal velocity (18 cm/s; Crucianelli et al., 2021) and consisted of six brief consecutive proximal-to-distal 9 cm-stimulations in each trial. Thus, participants rated the pleasantness of tactile stimuli in six different experimental conditions: the touch of the brush, the experimenter’s hand, and the stick at both slow and fast velocity.

As anticipated, participants provided ratings of both imagined and real tactile stimuli, in each condition, in two separated tasks. Participants completed the imagery task before the real one to avoid any contamination of the imagined pleasantness by the perceived pleasantness. The same experimenter, who was always a woman, administered both tasks in each group.

As previously described (Tagini et al., 2023), in the *imagery task* blindfolded participants imagined tactile stimuli on the back of their left forearm. Before the imagery procedure, participants saw six videoclips showing a female hand touching a paper cylinder as expected in each condition of stimulation (see Supporting Information in Tagini et al., 2023). Then, the experimenter guided participants through the imagery task by verbal instructions: “Please, close your eyes and keep them closed until further notice. Each time, I will tell which of the touch you should imagine; imagine this touch on your left forearm, the same way you saw it in the video, until you hear my stop signal. Then, tell me how pleasant this touch might be for you from zero—not pleasant at all—to 100—extremely pleasant.” Participants imagined the touch of each tool, at both velocities, only one time as done in our previous experiment (Tagini et al., 2023) and in Crucianelli et al. (2021): overall, the imagery task included six trials, randomized across participants.

In the real task, the experimenter touched the dorsal surface of participants’ forearm along a 9 cm washable black line aligned with their middle finger. Stimuli were delivered alternatively to the left and right of the line since tactile habituation could blunt sensory perception. Participants verbally judge each stimulus with the same scale previously used (i.e., from zero—not pleasant at all—to 100—extremely pleasant) and kept their eyes closed until the end of the procedure. Participants evaluated the pleasantness of touch in each condition of stimulation for three times in a pseudo-randomized order (i.e., with no consecutive repetitions of the same trial): overall, the experimental procedure included 18 trials. Participants judged each stimulus regardless previous repetitions.

#### 2.2.2. Psychological questionnaires

Participants completed the self-report questionnaires after the affective touch paradigm, to avoid any bias:

- The *Tactile Biography* questionnaire (TBIO) (Beltrán et al., 2020) evaluates the experience of affective touch in close relationships across the lifespan. This measure includes 29 items scored on a Likert-type scale ranging from 1 (e.g., not at all true for me) to 5 (e.g., extremely true), assessing four independent components: the frequency of and satisfaction for affective touch (*i*) in *childhood/adolescence* and (*ii*) *adulthood*, the overall (*iii*) *comfort* with, and (*iv*) *fondness* for interpersonal touch in close relationships. Higher scores indicate higher frequency/satisfaction, comfort, and fondness for affective touch. Three additional items record the (*v*) *feelings* experience in bodily affective interactions, (*vi*) the *presence of negative/unpleasant experiences* involving interpersonal touch, and (*vii*) the *preference* for giving and/or receiving affective touch.
- The *Anticipatory and Consummatory Interpersonal Pleasure Scale* (ACIPS) (Gooding and Pflum, 2014) measures the hedonic experience associated with common social and interpersonal scenarios through 17 items exploring individuals’ tendency to look forward to social interactions (anticipatory interpersonal pleasure—7 items) and to experience pleasure in social contexts (consummatory interpersonal pleasure—10 items). The ACIPS is scored on a Likert-type scale ranging from 1 (very false for me) to 6 (very true for me); higher scores indicate higher anticipatory and consummatory interpersonal pleasure.

### 2.3. Analyses

Preliminary analyses included the computation of overall descriptive statistics (i.e., means, standard deviations, and frequencies). Relative to continuous variables, the presence of possible univariate outliers (i.e., z-score > 2.5) and normality violations (according to skewness and kurtosis, Kim, 2013) was checked.

Independent sample *t*-tests were used to check for any differences between the two groups in terms of *Age*, *Years of Education*, and *Body Mass Index.*

Statistical analyses for the imagery and the real task were performed independently since the different number of trials included in the imagery (i.e., 6) and real (i.e., 18) task makes the experimental effects hardly comparable. Also, we could not counterbalance the sequence of the two tasks since the imagery task must precede the actual perception of stimuli; on the contrary, a direct comparison between the imagined and real pleasantness would require controlling for possible confounding effects related to the order of administration of the two tasks. For these reasons, including the data from the imagery and real tasks in the same statistical model may not methodologically grounded.

Thus, for both tasks, a mixed ANOVA was performed with *Group* (obesity *vs* healthy controls) as a between-subjects factor and *Speed* (affective *vs* non-affective) and *Tool* (brush *vs* hand *vs* stick) as within-subjects factors, to probe any difference between the groups in terms of the averaged pleasantness for affective touch-like stimulations. In case of a significant interaction, we performed *post hoc* multiple comparisons using estimated marginal means and applying Bonferroni’s correction. A critical two-tailed *p* ≤ 0.05 was adopted.

Psychological questionnaires were scored according to instructions reported in the seminal articles. Questionnaires ordinal scores were compared between the two groups with non-parametrical Mann–Whitney *U* tests. A one-tailed *p* ≤ 0.05 was considered significant since a directional hypothesis was formulated (i.e., reduced experience of affective touch in lifespan, and diminished interpersonal pleasure in women with obesity than healthy weight women).

Finally, we computed the affective touch sensitivity index relative to each tool in each task (i.e., imagery and real), as the difference between the pleasantness for affective and non-affective touch weighted by the overall pleasantness rating [i.e., (pleasantness for affective touch – pleasantness for non-affective touch)/∑ (pleasantness for affective touch, pleasantness for non-affective touch)/2] (Croy et al., 2016; Tagini et al., 2023). Then, we explored the possible associations between the affective touch sensitivity for the imagined and real touch, relative to each tool, the ACIPS score and the TBIO subscales scores by computing non-parametric Spearman’s coefficients of correlation separately in each group. We adopted one-tailed exact significant values since we expect lower affective touch sensitivity in case of higher level of social anhedonia and lower lifespan experience of affective touch. Bonferroni’s correction for multiple comparisons was applied.

### 2.4. Sample size calculation

*A priori* power analysis with G*Power software (Version 3.1) (Faul et al., 2007) was performed relative to the main aim of the study (i.e., the comparisons of pleasantness ratings reported in the experimental task, between groups and conditions). As mentioned, we plan to use a repeated-measures ANOVA with *Group* (obesity *vs* healthy controls) as a between-subjects factor and *Speed* (affective *vs* non-affective) and *Tool* (brush *vs* hand *vs* stick) as within-subjects factors. Assuming a correlation coefficient of 0.5 and medium effect size (*f* = 0.25), 14 participants in each group (28 participants, overall) would be required to reach a statistical power of 0.95, with alpha of 0.05.

## 3. Results

### 3.1. Participants

Fourteen women with obesity and 14 healthy controls were enrolled (Table 1).

**Table 1:** Demographic information and bodily characteristics of our sample.

	Obesity	Healthy weight	Statistical results			
	*n* = 14	*n* = 14	*t*	*df*	*p*-value	*d’*
Age	47.21 (15.54)	24.64 (3.25)	5.32	26	**0.001**	2.02
Education (years)	11.21 (2.75)	16.64 (1.86)	6.11	26	**0.001**	2.31
BMI (Kg/m^2^)	43.79 (4.09)	22.64 (2.42)	16.63	26	**0.001**	6.29

Data are expressed as means and standard deviations (in brackets). In bold, we report significant differences.

BMI = Body Mass Index, expressed as body mass (kg)/height (m^2^); weight and height were measured to the nearest 0.1 kg and 0.1 cm, respectively.

The obesity group showed older age and a lower level of schooling in comparison to the healthy controls, as registered in previous studies (Tagini et al., 2020a,b, 2021). As expected, participants with obesity had a higher BMI than controls.

### 3.2. Affective touch paradigm

#### 3.2.1. Preliminary analyses

Concerning the imagery task, one univariate outlier was found in the group of women with healthy weight^1^ relative to the *affective* touch of the hand; this participant was removed from the sample restoring the normality of distribution within the group. After the outlier removal, a small departure from normality, which is not expected to affect the robustness of the F statistic according to box plots and q-q plots inspection, emerged for both raw data and residuals relative to the *non-affective* touch of the brush. Also, we adopted Greenhouse–Geisser correction because of sphericity violation of the Tool*Speed two-way interaction (Mauchy’s test: *p* = 0.013). Non-homogeneity of variances according to Levene’s median tests was observed for the touch of the brush (*p* = 0.03) and the touch of the hand (*p* = 0.007) in the affective condition of stimulation; however, it was suggested that ANOVA results can still be considered robust if samples are similar in size (Kohr and Games, 1974).

Relative to the real task, no univariate outlier was detected (i.e., z-score > 2.5). Departure from normality of both raw data and residuals was observed concerning the *non-affective* touch of the hand; box plots and q-q plots inspection revealed a small departure from normality, which is not expected to affect the robustness of the F statistic. Homogeneity of variances was observed (Levene’s median tests *p* > 0.05); Greenhouse–Geisser correction was adopted due to a sphericity violation relative to the main effect of *Tool* and Tool*Speed two-way interaction, as assessed by Mauchy’s tests (*p* < 0.001).

#### 3.2.2. Imagery task

**Figure 1:**
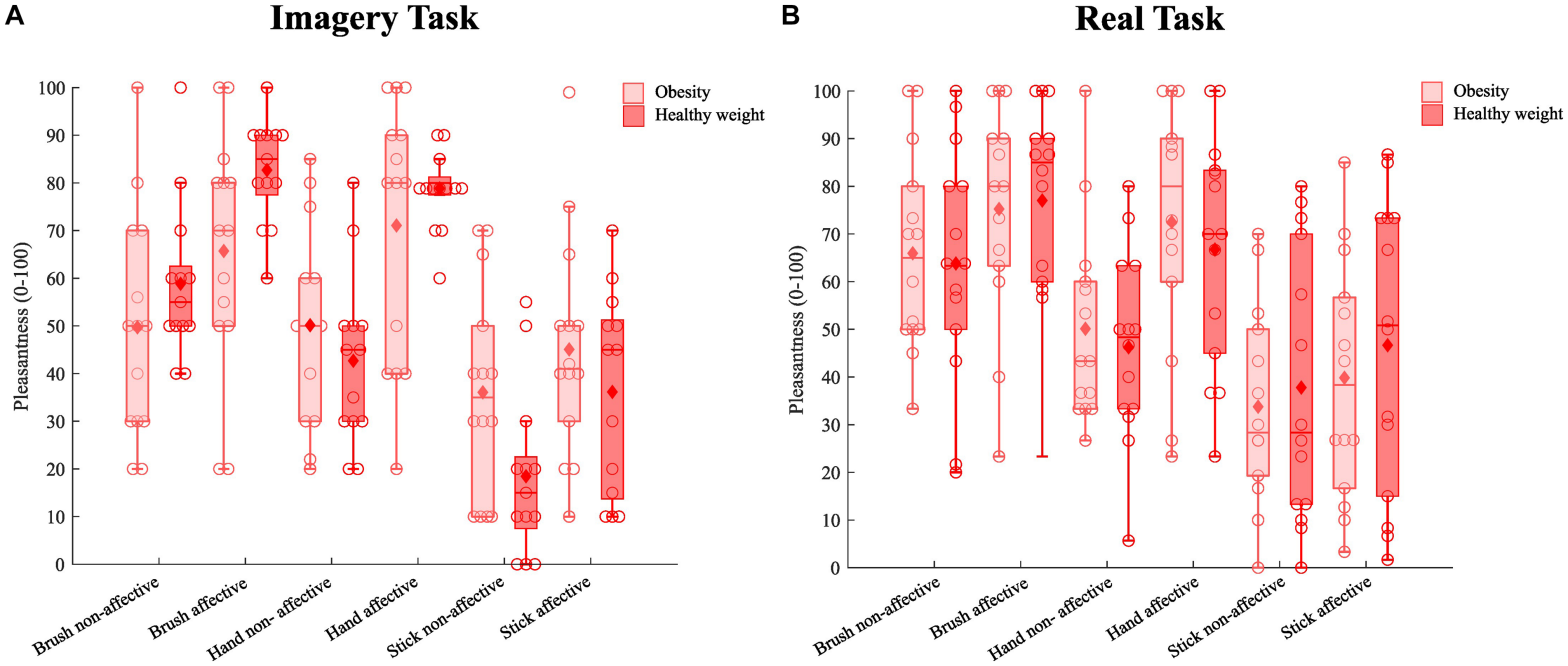
Boxplots illustrates the level of pleasantness experienced (y-axis) for affective and non-affective touch in both the imagery **(A)** and real task **(B)**, as reported by participants with obesity (pink) and participants with a healthy weight (red). Medians are conventionally reported as horizontal lines; diamonds indicate the average pleasantness of affective touch in each experimental condition (Tool*Speed*Group). Circles indicate the pleasantness reported by each participant in each condition (Tool*Speed*Group).

Means and standard deviations for each condition of stimulation in the imagery task are reported in Table 2 and Figure 1A.

**Table 2:** Level of pleasantness experienced in the case of affective and non-affective touch in both the imagery and real task, rated by participants with obesity and participants with a healthy weight.

		Imagery task	Real task		
		Obesity *n* = 14	Healthy weight *n* = 13	Obesity *n* = 14	Healthy weight *n* = 14
Brush	Affective	65.71 (25.10)	82.69 (12.12)	75.24 (22.93)	77.02 (21.98)
	Non-affective	49.71 (23.75)	58.84 (16.60)	65.95 (20.92)	63.81 (24.85)
Hand	Affective	71.07 (27.19)	78.84 (8.20)	72.50 (26.01)	66.74 (24.31)
	Non-affective	50.14 (20.63)	42.69 (17.98)	50.12 (20.72)	46.24 (19.84)
Stick	Affective	45.07 (23.39)	36.15 (21.22)	39.83 (24.73)	46.67 (30.68)
	Non-affective	36.07 (21.85)	18.46 (17.61)	33.76 (20.58)	37.85 (28.71)

Data are expressed as means and standard deviations (in brackets).

**Figure 2:**
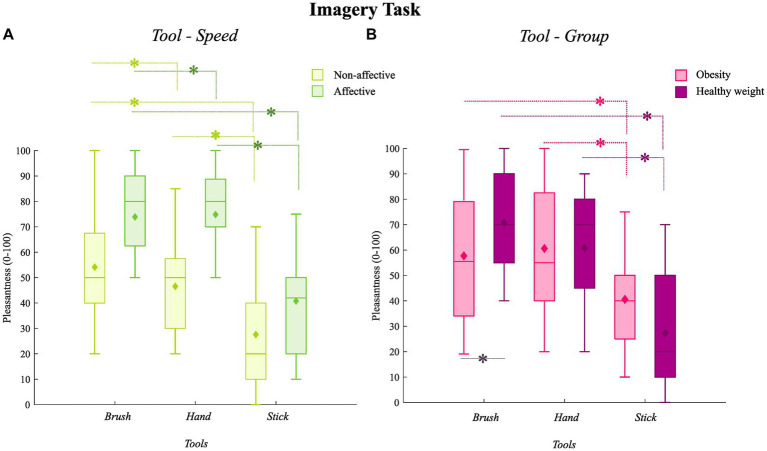
**(A)** Illustrates the Tool*Speed interaction for the imagery task. Boxplots illustrate the level of pleasantness experienced (y-axis) relative to the three tools (x-axis) in the affective (acid green) and non-affective (darker green) condition of stimulation. Medians are conventionally reported as horizontal lines; diamonds indicate the marginal means of the pleasantness of affective touch in each condition (Tool*Speed). **(B)** Illustrates the Tool*Group interaction for the imagery task. Boxplots illustrate the level of pleasantness experienced (y-axis) relative to the three tools (x-axis) in obesity (in pink) and health weight (purple). Medians are conventionally reported as horizontal lines; diamonds indicate marginal means of the pleasantness of affective touch in each condition (Tool*Speed).

A significant main effect of *Speed* (*F*_1,25_ = 47.63; *p* = 0.001, η^2^ = 0.66) emerged: overall the affective touch (marginal *M* = 63.26, SE = 3.19) was imagined as more pleasant than non-affective touch (marginal *M* = 42.66, SE = 2.68). Also, a significant main effect of *Tool* (*F*_2,50_ = 41.81, *p* = 0.001, η^2^ = 0.63) emerged: according to *post hoc* comparisons, the touch of the stick (marginal *M* = 33.94, SE = 3.47) was imagined as less pleasant than both the touch of the brush (marginal *M* = 64.24, SE = 2.90; *p* = 0.001, 95%CI [20.92, 26.68]) and the hand (marginal *M* = 60.69, SE = 3.44; *p* = 0.001, 95%CI [16.64, 36.86]); conversely, no difference emerged between the imagined touch of the brush and the hand (*p* = 0.85, 95%CI [−4.75, 11.86]). However, main effects should be read in the light of a significant Tool*Speed interaction (*F*_1.54, 38.4_ = 3.94, *p* = 0.037, η^2^ = 0.14): even though the affective touch was always judged as significantly more pleasant than the non-affective one, regardless the tool used for the stimulation, a qualitative inspection of Figure 2A and mean differences (see Table 3) suggests that the difference between the pleasantness for affective and non-affective touch was more pronounced when the touch was delivered with the hand than the other tools. Also, although the touch delivered by the brush and the hand were always rated as similarly pleasant, and as more pleasant than the touch of the stick, in both the affective and non-affective condition, the difference between the touch of the brush and the hand seems slightly more pronounced in the affective than non-affective condition of stimulation (see Table 4). Marginal means, standard errors, and statistics concerning *post hoc* comparisons are reported in Tables 3, 4.

**Table 3:** Marginal means (M Mean) and standard errors (SE) of the level of the imagined pleasantness relative to each tool in the affective and non-affective condition (i.e., Tool*Speed interaction) are reported.

Speed	Affective	Non-affective	Affective *vs* non-affective		
Tool	M Mean (SE)	M Mean (SE)	Δ Means (Δ SE)	*p*	95% CI
Brush	74.20 (3.78)	54.28 (3.97)	19.92 (5.15)	**0.001**	[09.33;30.52]
Hand	74.96 (3.93)	46.42 (3.74)	28.54 (3.38)	**0.000**	[21.59;35.50]
Stick	40.61 (4.30)	27.27 (3.84)	13.35 (4.27)	**0.004**	[04.55;22.14]

Bonferroni-corrected *post hoc* comparisons are showed: mean differences (Δ Means) and the relative standard errors (Δ SE), *p-*values and 95% Confidence Intervals (95% CI) are reported for each comparison. Significant differences are in bold.

**Table 4:** Marginal means (M Mean) and standard errors (SE) of the level of the imagined pleasantness relative to the affective and non-affective condition for each tool (i.e., Tool*Speed interaction) are reported.

Tool	Brush	Hand	Stick	Brush vs. hand			Brush vs. stick			Hand vs. stick		
Speed	M Mean (SE)	M Mean (SE)	M Mean (SE)	Δ Means (Δ SE)	*p*	95% CI	Δ Means (Δ SE)	*p*	95% CI	Δ Means (Δ SE)	*p*	95% CI
Affective	74.20 (3.78)	74.96 (3.93)	40.61 (4.30)	−0.76 (2.05)	1.00	[−6.01;4.50]	33.59 (4.88)	**0.001**	[21.07;46.11]	34.35 (5.08)	**0.001**	[21.31;47.39]
Non-affective	54.28 (3.97)	46.42 (3.73)	27.26 (3.84)	7.86 (5.44)	0.48	[−6.10;21.83]	27.014 (5.03)	**0.001**	[14.10;39.93]	19.15 (3.75)	**0.001**	[9.54;28.76]

Bonferroni-corrected *post hoc* comparisons are showed: mean differences (Δ Means) and the relative standard errors (Δ SE), *p-*values and 95% Confidence Intervals (95% CI) are reported for each comparison. Significant differences are in bold.

No significant main effect of *Group* was found (*F*_1,25_ = 0.001, *p* = 1, η^2^ = 0.001; obesity marginal *M* = 52.96, SE = 3.51; healthy controls marginal *M* = 52.95, SE = 3.65). However, the Tool*Group interaction was significant (*F*_2,50_ = 6.60, *p* = 0.003, η^2^ = 0.21), indicating that women with obesity rated the imagined touch delivered by the brush as significantly less pleasant that women with healthy weight (see Figure 2B) but no significant differences emerged between the two groups for the pleasantness of the touch delivered by the experimenter’s hand and the stick. Marginal means, standard errors, and statistics concerning *post hoc* comparisons are reported in Tables 5, 6. The interactions Speed*Group (*F*_1,25_ = 3.15, *p* = 0.08, η^2^ = 0.11) and Tool*Speed*Group (*F*_1.54, 38.4_ = 2.78, *p* = 0.7, η^2^ = 0.01) were not significant. Thus, in line with Crucianelli et al. (2021), we observed that affective touch was perceived as more pleasant than non-affective touch even though tactile stimuli were just *imagined*, regardless of the group. However, we observed that this difference was enhanced when the touch was delivered by a human’s hand rather than by inanimate tools. In fact, the level of pleasantness reported seems related to the tool used: only the pleasantness experienced for the imagined touch of the brush was significantly lower in obesity than in healthy controls.

**Table 5:** Marginal means (M Mean) and standard errors (SE) of the level of imagined pleasantness relative to each tool in obesity and healthy weight controls (i.e., Tool*GR interaction) are reported.

Group	Obesity	Healthy weight	Obesity vs. Healthy weight		
Tool	M Mean (SE)	M Mean (SE)	Δ Means (Δ SE)	*p*	95% CI
Brush	57.71 (4.03)	70.77 (4.18)	−13.06 (5.80)	**0.033**	[−25.00;-1,11]
Hand	60.61 (4.78)	60.77 (4.96)	−0.16 (6.89)	0.981	[−14.35;14.02]
Stick	40.57 (4.82)	27.31 (5.00)	−13.26 (6.95)	0.068	[−01.05;27.57]

Bonferroni-corrected *post hoc* comparisons are showed: mean differences (Δ Means) and the relative standard errors (ΔSE), *p-*values and 95% Confidence Intervals (95% CI) are reported for each comparison. Significant differences are in bold.

**Table 6:** Marginal means (M Mean) and standard errors (SE) of the level of the imagined pleasantness relative to each tool in obesity and healthy weight controls (i.e., Tool*GR interaction) are reported.

Tool	Brush	Hand	Stick	Brush vs. hand			Brush vs. stick			Hand vs. stick		
Group	M Mean (SE)	M Mean (SE)	M Mean (SE)	Δ Means (Δ SE)	*p*	95% CI	Δ Means (Δ SE)	*p*	95% CI	Δ Means (Δ SE)	*p*	95% CI
Obesity	57.71 (4.03)	60.61 (4.78)	40.57 (4.82)	2.89 (4.49)	1.00	[−14.42;8.63]	17.14 (5.10)	**0.007**	[4.14;30.16]	2.89 (4.49)	**0.004**	[−8.63;14.42]
Healthy weight	70.77 (4.18)	60.77 (4.96)	27.31 (5.00)	10.00 (4.66)	0.13	[−1.96; 21.96]	43.46 (5.26)	**0.001**	[29.95;56.97]	33.46 (5.68)	**0.001**	[18.98;48.03]

Bonferroni-corrected *post hoc* comparisons are showed: mean difference (Δ Means) and the relative standard errors (ΔSE), *p-*values and 95% Confidence Intervals (95% CI) are reported for each comparison. Significant differences are in bold.

#### 3.2.3. Real task

Means and standard deviations for each condition of stimulation in the real task are reported in Table 2 and Figure 1B.

**Figure 3:**
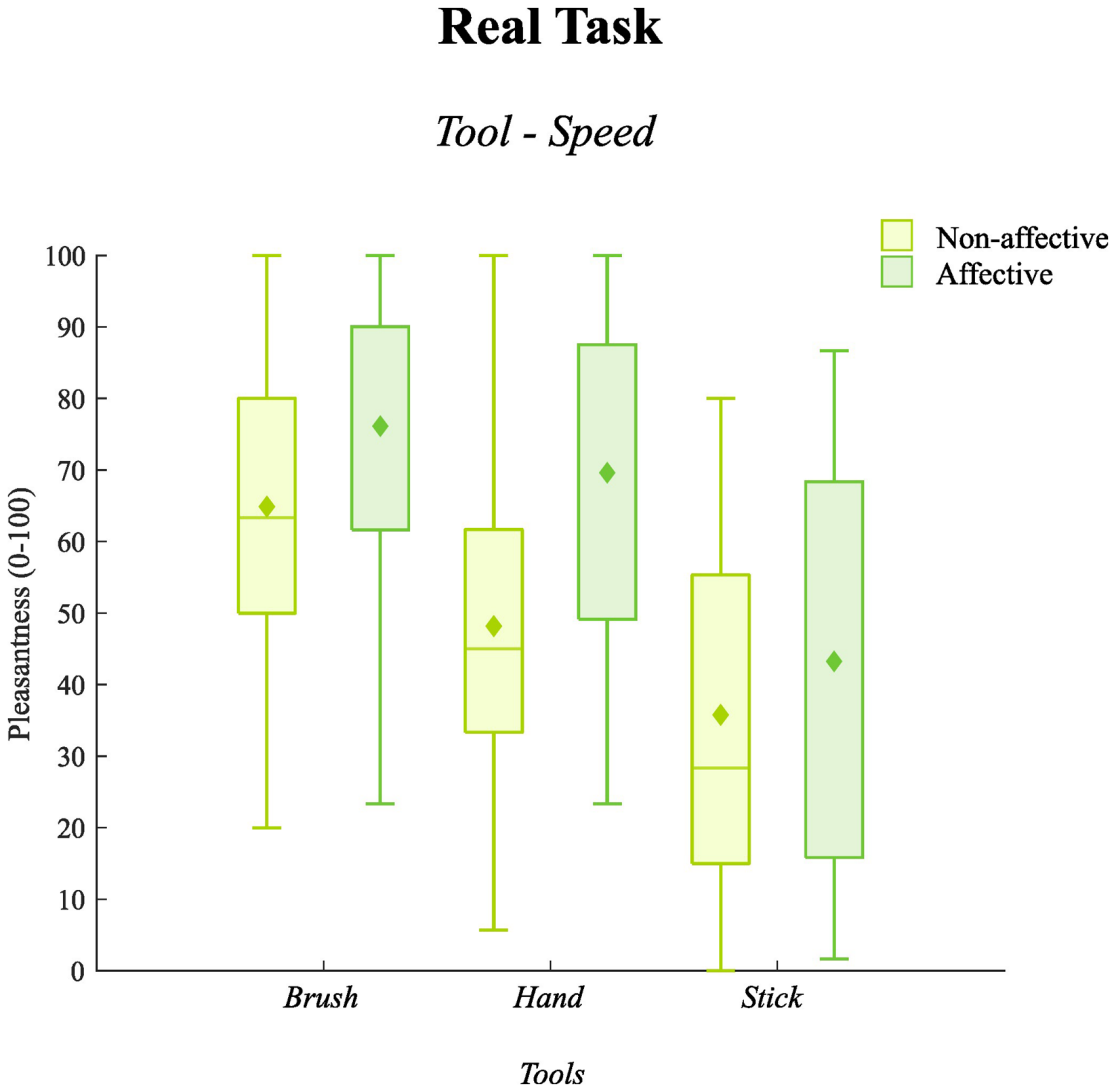
The Tool*Speed interaction for the real task is illustrated. Boxplots represent the level of pleasantness experienced (y-axis) relative to the three tools (x-axis) in the affective (acid green) and non-affective (darker green) condition of stimulation. Medians are conventionally reported as horizontal lines; diamonds indicate the marginal means of the pleasantness of affective touch in each condition (Tool*Speed). As reported in Tables 7, 8 each comparison was statistically significant (*p* < 0.05).

A significant main effect of *Speed* emerged (*F*_1,26_ = 15.20, *p* = 0.001, η^2^ = 0.37): as expected, affective touch (marginal *M* = 63, SE = 3.92) was more pleasant than non-affective touch (marginal *M* = 49.61, SE = 3.49). Also, a significant main effect of *Tool* (*F*_1.17, 30.49_ = 32.67, *p* = 0.001, η^2^ = 0.56) emerged suggesting that, overall, the touch of the brush (marginal *M* = 70.51, SE = 3.73) was more pleasant than both the touch of the hand (marginal *M* = 58.90, SE = 3.41; *p* = 0.001, 95%CI [7.60, 15.62]) and the stick (marginal *M* = 39.51, SE = 4.69; *p* = 0.001, 95%CI [18.90, 44.09]) and the touch of the hand was more pleasant than the touch of the stick (*p* = 0.001, 95%CI [7.87, 30.90]). However, the Tool*Speed interaction was significant (*F*_1.34, 34.96_ = 4.56, *p* = 0.03, η^2^ = 0.15), suggesting that the mentioned difference between the touch of the hand and the stick was statistically relevant only in the affective condition of stimulation (see Figure 3). Marginal means, standard errors, and statistics concerning *post hoc* comparisons are reported in Tables 7, 8.

**Table 7:** Marginal means (M Mean) and standard errors (SE) of the level of the real pleasantness relative to each tool in the affective and non-affective condition (i.e., Tool*Speed interaction) are reported.

Speed	Affective	Non-affective	Affective *vs* non-affective		
Tool	M Mean (SE)	M Mean (SE)	Δ Means (ΔSE)	*p*	95% CI
Brush	76.13 (4.25)	64.88 (4.34)	11.25 (4.25)	**0.014**	[2.51;19.98]
Hand	69.62 (4.76)	48.18 (3.83)	21.44 (5.30)	**0.001**	[10.55;32.33]
Stick	43.25 (5.27)	35.77 (4.72)	7.48 (3.49)	**0.042**	[0.31;14.65]

Bonferroni-corrected *post hoc* comparisons are showed: mean differences (Δ Means) and the relative standard errors (ΔSE), *p-*values and 95% Confidence Intervals (95% CI) are reported for each comparison. Significant differences are in bold.

**Table 8:** Marginal means (M Mean) and standard errors (SE) of the level of real pleasantness relative to the affective and non-affective condition for each tool (i.e., Tool*Speed interaction) in are reported.

Tool	Brush	Hand	Stick	Brush vs. hand			Brush vs. stick			Hand vs. stick		
Speed	M Mean (SE)	M Mean (SE)	M Mean (SE)	Δ Means (Δ SE)	*p*	95% CI	Δ Means (Δ SE)	*p*	95% CI	Δ Means (Δ SE)	*p*	95% CI
Affective	76.13 (4.25)	69.62 (4.76)	43.25 (5.27)	6.51 (1.68)	**0.002**	[2.21;10.81]	32.88 (5.52)	**0.001**	[18.75;47.01]	26.37 (5.77)	**0.001**	[11.62;41.12]
Non-affective	64.88 (4.34)	48.18 (3.83)	35.77 (4.72)	16.70 (2.38)	**0.001**	[−6.10;21.83]	29.11 (5.32)	**0.001**	[15.49; 42.73]	12.41 (4.88)	**0.052**	[−0.09;24.90]

Bonferroni-corrected *post hoc* comparisons are showed: mean differences (Δ Means) and the relative standard errors (Δ SE), *p-*values and 95% Confidence Intervals (95% CI) are reported for each comparison. Significant differences are in bold.

The main effect of *Group* (*F*_1,26_ = 0.001, *p* = 0.98, η^2^ = 0.001) was not significant: indeed, the obesity group (marginal *M* = 56.23, SE = 4.65) and healthy controls group (marginal *M* = 56.38, SE = 4.65) reported the same level of experienced pleasure for the tactile stimulations.

Finally, the Tool*Group (*F*_1.17, 30._49 = 0.88, *p* = 0.37, η^2^ = 0.03), Speed*Group (*F*_1,26_ = 0.056, *p* = 0.82; η^2^ = 0.002), and Tool*Speed*Group (*F*_1.34, 34.96_ = 0.21, *p* = 0.72, η^2^ = 0.01) interactions were not significant.

As expected, and in line with the previous literature in the field (Löken et al., 2009; Gordon et al., 2013), the pleasantness of affective touch was higher than non-affective touch, regardless the tool used. However, we observed that participants with obesity and healthy weight perceived comparable pleasantness of both affective and non-affective touch, in contrast with our hypothesis.

### 3.3. Psychological questionnaires

#### 3.3.1. Affective touch experience across lifespan

Table 9 illustrates means and standard deviations for the TBIO questionnaire scores in each group.

**Table 9:** Means and standard deviations (in brackets) for the subscales of the *Tactile Biography questionnaire* (TBIO) and the total score of the *Anticipatory and Consummatory Pleasure Scale* (ACIPS) relative to participants with obesity and healthy weight; *p-*values refer to the between-groups comparisons performed by Mann Whitney *U*-tests based on mean ranks.

	Obesity	Healthy weight	*p*
Tactile Biography Questionnaire			
Childhood/adolescence	3.17 (0.91)	3.92 (0.90)	**0.02**
Comfort	4.00 (0.97)	3.77 (0.80)	0.20
Fondness	4.01 (0.86)	4.07 (1.02)	0.34
Anticipatory and Consummatory Pleasure Scale^♦^			
Total score	84.08 (11.88)	87.5 (10.11)	0.14

We report significant differences in bold. ^♦^ACIPS total score of one participant with obesity was missing.

The *childhood/adolescent affective touch experiences* score was significantly lower (*U* = 53.5, *z* = −2.05, *p* = 0.02; *r* = 0.39) in the obesity (mean rank = 12.32) than in healthy controls (mean rank = 17.68), in line with our prediction about lower early exposure to affective touch in obesity. On the contrary, no differences between the two groups were found for the *adulthood affective touch experiences* score (*U* = 81.5, *z* = −0.76, *p* = 0.23, *r* = 0.14; obesity mean rank = 13.32, healthy controls mean rank = 15.68), the *fondness* score (*U* = 88.50, *z* = −0.44, *p* = 0.34, *r* = 0.08; obesity mean rank = 13.82, healthy controls mean rank = 15.18), and the *comfort* score (*U* = 79.50, *z* = −0.85, *p* = 0.20, *r* = 0.16; obesity mean rank = 15.82, healthy controls mean rank = 13.18). Results concerning the three additional items of the TBIO questionnaire are reported in Supplementary Table S1 supplementary analyses showed that there were no differences between the two groups concerning previous negative experiences involving interpersonal touch, preference for giving and/or receiving affective touch, or the prevalence of specific emotions and sensations related to affective touch.

**Figure 4:**
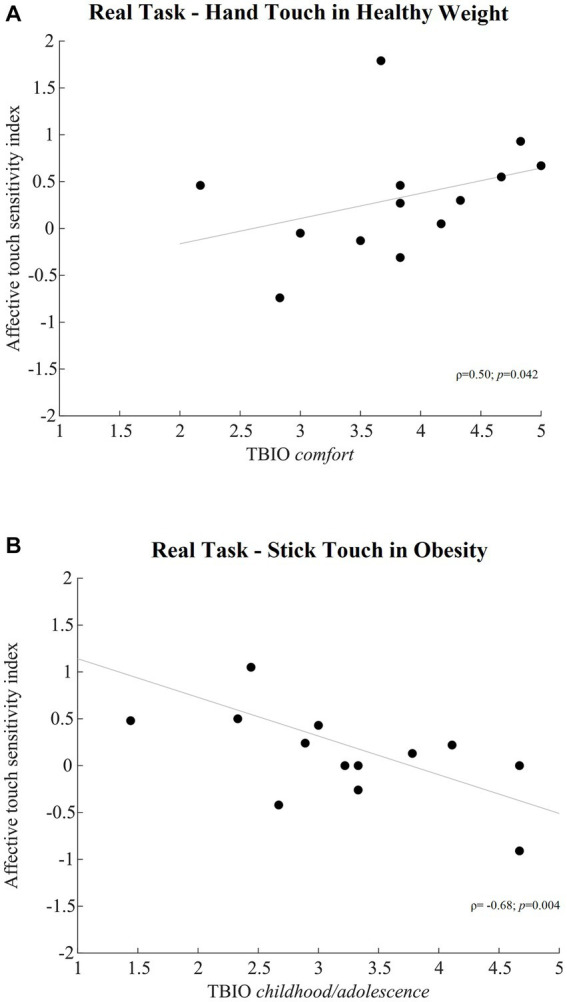
**(A)** Shows the relationship between the affective touch sensitivity index for the real touch of the hand (y-axis) and the *comfort* subscale of the Tactile Biography (i.e., TBIO) questionnaire (x-axis) in healthy individuals. **(B)** Shows the relationship between the affective touch sensitivity index relative to the real touch of the stick (y-axis) and the *childhood/adolescence* subscale of the Tactile Biography (i.e., TBIO) questionnaire (x-axis) in participants with obesity. ρ, Spearman’s coefficient of correlation; *p*, one-tailed statistical significance.

The computation of Spearman’s coefficients of correlation between the affective touch sensitivity index, relative to the three tools in both the imagery and real task and in each group, and the TBIO subscales scores suggest a positive significant correlation between the affective touch sensitivity index relative to the real touch of the hand and the TBIO *comfort* score (*ρ* = 0.50; *p* = 0.042) in the group of women with healthy weight (Figure 4A). That is, the higher the comfort for affective touch in real life, the higher was the preference for the affective than the non-affective touch of the experimenter’s hand. On the other hand, in our participants with obesity (Figure 4B) the affective touch sensitivity index relative to the real touch of the stick significantly and negatively correlated with the TBIO *childhood/adolescent affective touch experiences* score (*ρ* = − 0.68; *p* = 0.004), suggesting that the lower was the frequency and satisfaction for the early experience of intimate contact the higher was the preference for the slow than the fast touch of the stick. No other significant correlation emerged (Table 10).

**Table 10:** Spearman’s coefficient of correlation between the in the imagery and real tasks, relative to the touch of the brush, the hand and the stick, the *Tactile Biography* (TBIO) *questionnaire* subscales, and the total score of the *Anticipatory and Consummatory Pleasure Scale* (ACIPS) in each group.

		TBIO						ACIPS^♦^	
		Childhood/adolescence		Comfort		Fondness		Total score	
		Obesity	Healthy weight	Obesity	Healthy weight	Obesity	Healthy weight	Obesity	Healthy weight
Imagery task	Brush	0.40	−0.19	0.15	−0.05	0.12	−0.21	−0.28	−0.42
	Hand	−0.30	0.05	0.11	−0.11	−0.12	−0.01	−0.05	−0.22
	Stick	0.07	−0.15	0.46	−0.30	0.45	−0.44	0.41	−0.10
Real task	Brush	−0.16	−0.14	−0.17	0.37	−0.09	−0.08	−0.46	0.02
	Hand	−0.38	−0.07	−0.20	**0.50**	−0.13	−0.05	−0.24	0.06
	Stick	**−0.68**	0.01	−0.21	0.47	−0.28	−0.17	0.10	0.15

Significant correlations are reported in bold (one-tailed *p* < 0.05); ^♦^ ACIPS total score of one participant with obesity was missing.

#### 3.3.2. Social anhedonia

Table 9 illustrates means and standard deviations for the ACIPS total score in each group. The mean rank total score was not significantly different between the two groups (*U* = 68.5, *z* = −1.09, *p* = 0.14, *r* = 0.20; obesity mean rank = 12.27, healthy controls mean rank = 17.68), suggesting a similar anticipatory and consummatory interpersonal pleasure (i.e., the hedonic experience associated with the social contexts) in our women with obesity and with healthy weight.

We observed no significant correlation between the affective touch sensitivity index, relative to the three tools, in both the imagery and real task and the ACIPS total score, separately in each group (Table 10).

## 4. Discussion

In our knowledge, no previous study investigated affective touch in obesity; however, the exploration of this topic may shed light on the possible association between the perception of bodily sensory information (i.e., affective tactile sensations) and the experience of unsatisfactory intimate relationships in this clinical condition, which is characterized by low satisfaction and psychological distress.

Our results suggest a preserved experience of affective touch in obesity: indeed, participants with obesity and healthy weight reported a similar level of pleasantness for tactile stimulations recalling affection. Crucially, this similarity emerged also when our participants imagined receiving an affective touch, in absence of any real tactile stimulation on the skin. These results are surprising; however, they are consistent with the patients’ *subjective* hedonic experience of affective touch and social interactions in adulthood. Our participants with obesity reported to enjoy and appreciate interpersonal relationships and affective bodily interactions in real life, as much as women with healthy weight. Conversely, they described a reduced experience of affective interpersonal contacts in childhood and adolescence. In other words, our results do not support the hypothesis of an association between a limited history of past affective bodily contacts and an altered experience of affective touch in adulthood (Beltrán et al., 2020). Furthermore, these findings seemed difficult to reconcile with the evidence suggesting that individuals with obesity experience inter-social difficulties (Albano et al., 2019). In fact, our results suggest that individuals with obesity appreciate and are willing to be in relation to other people.

How could we interpret this unexpected observation? The *social reconnection hypothesis* suggests that the experience of social rejection elicits the desire to renew affiliative bonds, promoting prosocial behaviors and the creation of new relationships (Maner et al., 2007). That is, early and past experiences of dismissing caring relationships, at least in terms of physical interactions, may have invigorated individuals’ desire of more satisfying relationships, despite the intrapsychic conflicts and the multiple difficulties experienced during social interactions (Albano et al., 2019). Therefore, in line with the interpretation of our results in terms of a *social reconnection* (Maner et al., 2007), we suggest that individuals with obesity can experience adequate intimacy and pleasure in significant relationships when mediated by bodily contacts. Furthermore, concerning the secondary aim of this study, we observed that the more our participants with healthy weight are comfortable with interpersonal and intimate contact in their everyday life, the higher was their preference for the affective, over the non-affective, touch of the experimenter’s hand. Unsurprisingly, this evidence suggests that healthy individuals who are, overall, more at ease with interpersonal bodily contact may appreciate more the gentle touch of a stranger in the experimental setting. On the other hand, the lower the experience of early intimate contact, the higher was the preference of participants with obesity for the affective than non-affective touch of the stick. Interpreting this, unexpected, observation is especially challenging since the touch of the stick was a control condition of stimulation, meaning that it should not vehicle any pleasure. On the methodological side, we may note that our correlational analyses might not have sufficient power since power analyses and sample size were computed considering the comparison of affective touch between the two groups (i.e., the primary aim of the study). On the other hand, lack of previous evidence relative to affective touch in obesity make any speculation quite a long shot, encouraging future investigations to probe further this field of study. In fact, we suggest that multiple, but unexplored aspects may affect the experience of bodily contact in social or intimate relationships. First, the experience of bodily interactions depends on the characteristics of the other person involved. In real life affective touch involves loved ones; on the contrary, in the experimental settings, the affective touch stimulation (i.e., a specific tactile bodily interaction used to communicate intimacy) was delivered by the experimenter, who was unfamiliar and meaningless to the participants. Indeed, we recognize that this scenario might not be ideal since individuals may even perceive the touch of a stranger as unpleasant, undesired, and disagreeable, as well as a violation of bodily space. Accordingly, it may be questioned whether adopting a different experimental perspective, in which someone who is familiar to the participant delivers the tactile stimulation, would lead to a different result. Also, in the context of obesity, it may be interesting to investigate the possible effect of the other’s bodily physical appearance. Considering that people with obesity often experience weight-related stigmatization, especially by people who are not overweight (Albano et al., 2019), one may expect them to feel less comfortable when touched by someone with a dissimilar weight-status. Affective touch imagery paradigms, which we demonstrated to be comparable to traditional in-person procedures, may be adopted to manipulate *who* delivers the touch, recreating more veridical scenarios. Also, the evidence of a comparable experience of affective touch in the imagery and real task supports the role of top-down cognitive processes (Sailer and Leknes, 2022), beyond bottom-up sensory mechanisms, in the experience of affective bodily contact.

As anticipated, this study is unique since it represents the first investigation of affective touch in obesity. However, the study limitations should be underlined. First, only female participants were recruited to guarantee a match with the experimenter’s gender, preventing possible confounding effects (e.g., more embarrassment or a higher sexual connotation of touch). Still, our conclusions may be hardly generalized to men since the perception of affective touch is significantly affected by gender (Russo et al., 2020). In future research, imagery procedures may beat this limitation, not only matching individuals’ gender more easily but also deliberately creating different “gender pairs” to verify how the experience of affective touch may differ across different affective relationships (e.g., with partners, primary caregivers, friends). However, a measure of vividness of the imagined touch (e.g., with a visual analog scale) should be included since individuals with obesity may find it more difficult to imagine affective touch, considering the limited experience of intimate interactions. Furthermore, our results should be cautiously generalized across sociocultural and ethnical backgrounds. Distinct cultures have singular approaches to caring and different social norms regulating interpersonal contact; thus, cultural differences might influence individuals’ predisposition to bodily interactions and the amount of experience of interpersonal touch across lifespan (Schirmer et al., 2022). Our participants were all Caucasian with the only exception of one Moroccan woman; therefore, we believe this aspect hardly affected our results. Nonetheless, the relationship between one’s sociocultural and ethnical background and the perception of affective touch represents an intriguing topic, which deserve broaden investigation.

From a methodological perspective, we note that participants with obesity on average were older and had a lower educational level than healthy weight participants. We do not expect the educational level to have any effect on the affective touch perception; also, the task and the questionnaires were especially clear and simple, so that the educational level should not affect participants’ understanding and responses. Regarding age, a recent systematic review (Cruciani et al., 2021) showed that the explicit preference for slow (i.e., CT-optimal) over a fast (i.e., CT- non-optimal) touch seems preserved across different ages, despite the overall lack of solid evidence on how affective touch may be modulated in people older than 40 years. However, since the perceived pleasantness for a gentle stroking seems to increase with age (Sehlstedt et al., 2016) patients’ older age might have concealed any significant difference between the two groups. On the other hand, we guaranteed that women with obesity and women with healthy weight have no health-related issues that may influence the perception of affective touch, such as neuropathic pain, psychiatric diseases, or personality disorders; however, we should note that women with obesity might be more at risk of developing physical and psychological comorbidities, such as cardiovascular, metabolic, respiratory, and osteoarticular problems, or dysfunctional eating behaviors, whose possible interplay with the perception of affective touch in obesity is unknow.

Additionally, individuals’ experience of affective touch and interpersonal relationships might depend on whether obesity is recent or long-lasting. There is evidence suggesting that typically obesity is a lifetime clinical condition (Simmonds et al., 2016); indeed, in clinical practice, affected individuals often cannot report the specific onset of the disorder, unless it is secondary to other conditions (e.g., drugs or other pathologies). Instead, they commonly recognize a considerable time window as onset, which is often back in time, such as childhood or adolescence. Future studies may probe whether the onset of obesity modulates the experience of affective touch, at least distinguishing between childhood/adolescence and adulthood onsets.

To conclude, we would bring the readership attention back to the evidence that although we observed a preserved experience of affective touch in obesity in adulthood, affected individuals claimed dismissing primary caring relationships, at least concerning physical interactions. Preliminary evidence suggests that childhood and adolescent experiences of affective touch are significant predictors of individuals’ style of attachment (Beltrán et al., 2020), which in turn influences how people interact with others (Simpson and Rholes, 2012). On the other hand, interpersonal difficulties play a role in both the onset and maintenance of obesity by motivating overeating behaviors (Ivanova et al., 2015; Lo Coco et al., 2016). A deeper understanding of the factors related to interpersonal difficulties in obesity may lead to better weight-related outcomes (Yorgason et al., 2019; Pratt et al., 2021); thus, future research may deepen the possible interplay between both early and adult experiences of affective touch (focusing on “ecological” scenarios), attachment style, and interpersonal difficulties in this clinical condition.

## Data availability statement

The datasets presented in this study can be found in online repositories. The names of the repository/repositories and accession number(s) can be found at: https://doi.org/10.5281/zenodo.7468106.

## Ethics statement

The studies involving humans were approved by the IRCCS Istituto Auxologico Italiano Committee. The studies were conducted in accordance with the local legislation and institutional requirements. The participants provided their written informed consent to participate in this study.

## Author contributions

ST: conceptualization, methodology, investigation, data curation, formal analysis, visualization, and writing—original draft. MS and AM: resources and writing—review and editing. FS: conceptualization, methodology, validation, project administration, supervision, and writing—review and editing. All authors contributed to the article and approved the submitted version.

## Funding

This research was partially funded by the Italian Ministry of Health.

## Conflict of interest

The authors declare that the research was conducted in the absence of any commercial or financial relationships that could be construed as a potential conflict of interest.

## Publisher’s note

All claims expressed in this article are solely those of the authors and do not necessarily represent those of their affiliated organizations, or those of the publisher, the editors and the reviewers. Any product that may be evaluated in this article, or claim that may be made by its manufacturer, is not guaranteed or endorsed by the publisher.

## Footnotes

^1^This participant did not differ in terms of BMI (*p* = 0.80) and TBIO childhood/adolescent affective touch experiences score (*p* = 0.95) from other healthy participants; however, she reported significantly lower scores on the comfort (*p <* 0.001) and fondness (*p <* 0.001) subscale of the TBIO questionnaire and in the ACIPS (*p <* 0.001).
