# Supplementary material for: The perception of affective touch in women affected by obesity
Source: Front Psychol. 2023 Aug 28;14:1171070. doi: 10.3389/fpsyg.2023.1171070 (PMC10493281; doi:10.3389/fpsyg.2023.1171070)
Supplement: Supplementary file 1 [file Table_1.pdf]

Supporting Information

S1 Tactile Biography (TBIO) questionnaire: Additional analyses and results

Three additional items exploring the lifespan experience of affective bodily contacts are included at the end of the TBIO questionnaire, measuring *i*) negative/unpleasant experiences involving interpersonal touch (yes / no), *ii*) the preference for giving or receiving affective touch (where answer options were: giving affective touch / receiving affective touch / both receiving and giving affective touch /neither giving or receiving affective touch), and *iii*) the specific sensations experienced in affective bodily contacts (where answer options were: relax / embarrassment / satisfaction / happiness / rejection / disgust / comfort / annoyance / uneasiness / pleasure).
The chi-square test was used to investigated whether the *Group* (obesity *vs* healthy weight) was significantly associated with the occurrence of specific answers. Two-sided, exact, p values ≤ 0.05 were considered statistically significant. Overall, no significant association with the group was observed: percentages and chi-squared statistics are reported in Table S1.

**TBIO additional items**		**Obesity**	**Healthy weight**
Negative experiences with interpersonal touch	χ^2=2.82, p=0.25, V=0.32		
yes		50% (7)	28.6% (4)
Preference for giving/receiving affective touch	χ^2=4.09, p=0.32, V=0.38		
giving		7.1 % (1)	14.3 % (2)
receiving		14.3% (2)	35.7 % (5)
both		78.6% (11)	42.9% (6)
neither of them		0% (0)	7.1% (1)
Feelings related to interpersonal touch			
relax	χ^2=2.19, p=0.33, V=0.28	71.4% (10)	92.9% (13)
embarrassment	χ^2=0.7, p=0.68, V=0.16	21.4% (3)	35.7% (5)
satisfaction	χ^2=0.7, p=0.68, V=0.16	37.5% (5)	50% (7)
happiness	χ^2=0.001, p=1, V=0.001	57.1% (8)	57.1% (8)

rejection	n/a	0% (0)	0% (0)
disgust	n/a	0% (0)	0% (0)
comfort	χ^2=2.8, *p*=0.21, *V*=0.32	57.1% (8)	85.7% (12)
annoyance	n/a	0% (0)	0% (0)
uneasiness	χ^2=0.37, *p*=1, *V*=0.12	14.3% (2)	7.1% (1)
pleasure	n/a	0% (0)	0% (0)
